# Obsessive-Compulsive Disorder with Suicide Obsessions in a First Responder without Previous Diagnosis of OCD or History of Suicide Attempts

**DOI:** 10.1155/2017/4808275

**Published:** 2017-10-02

**Authors:** Vivekananda Rachamallu, Michael M. Song, Haiying Liu, Charles L. Giles, Terry McMahon

**Affiliations:** ^1^Texas Tech University Health Sciences Center School of Medicine, Department of Psychiatry, Lubbock, TX, USA; ^2^Texas Tech University Health Sciences Center School of Medicine and Graduate School of Biomedical Sciences, MD/PhD Program, Lubbock, TX, USA; ^3^Texas Tech University Health Sciences Center School of Medicine, Lubbock, TX, USA

## Abstract

Obsessive-compulsive disorder (OCD) is a distressing and often debilitating disorder characterized by obsessions, compulsions, or both that are time-consuming and cause impairment in social, occupational, or other areas of functioning. There are many published studies reporting higher risk of suicidality in OCD patients, as well as studies describing increased risk of suicidality in OCD patients with other comorbid psychiatric conditions such as major depressive disorder (MDD) and posttraumatic stress disorder (PTSD). Existing case reports on OCD with suicide as the obsessive component describe patients with long standing diagnosis of OCD with suicidal ideations or previous suicide attempts. This report describes the case of a 28-year-old male, who works as a first responder, who presented with new onset symptoms characteristic of MDD and PTSD, with no past history of OCD or suicidality who developed OCD with suicidal obsessions. Differentiating between suicidal ideation in the context of other psychiatric illnesses and suicidal obsessions in OCD is critical to ensuring accurate diagnosis and timely provision of most appropriate treatment. The combination of exposure and response prevention therapy and pharmacotherapy with sertraline and olanzapine was effective in helping the patient manage the anxiety and distress stemming from the patient's OCD with suicidal obsession.

## 1. Introduction

The cardinal features of obsessive-compulsive disorder (OCD) include obsessions, compulsions, or both that are time-consuming and cause clinically significant distress or impairment in social, occupational, or other areas of functioning [1]. Obsessions are repetitive, intrusive, and unwanted thoughts or images (often of sexual, religious, aggressive, or death-related nature) that cause significant anxiety and distress [2]. Compulsions or rituals are repetitive behaviors or mental acts that are performed by those with OCD, in an attempt to decrease their anxiety or distress [1]. There are many published studies reporting higher risk of suicidality in OCD patients, as well as studies describing increased risk of suicidality in OCD patients with other comorbid psychiatric conditions such as major depressive disorder (MDD) and posttraumatic stress disorder (PTSD) [3–11]. Furthermore, violent obsessions have been reported to be positively correlated with suicidality in OCD [9, 12]. However, OCD with ego-dystonic suicidal obsessions have rarely been reported [13–15]. Al-Zaben described a patient with long history of both MDD and OCD who developed suicidal obsessions [13] while Wetzler et al. described the development of OCD with suicidal obsessions in a patient with MDD, following a suicide attempt [14]. Furthermore, Wetterneck et al. described OCD with suicidal obsession in a patient with treatment-resistant OCD [15] (see Table 1). This report describes the case of a 28-year-old male, who works as a first responder, who presented with symptoms characteristic of MDD and PTSD, with no past history of OCD or suicidality, with newly developed OCD with suicidal obsessions.

## 2. Case Description

A 28-year-old Caucasian male was brought to the emergency department (ED) by his wife and parents due to sudden onset, intense suicidal ideations. He had no significant past medical or psychiatric history except for a 6-month history of depressed mood and anxiety in the context of several stressors in his family and at work. The family reported that the patient developed blunted affect and significantly reduced vocalization over those 6 months. He did not have any prior history of suicide attempts or psychiatric diagnoses or hospitalizations. He reported drinking 3 beers up to 4 times a week but he denied any other history of substance abuse or any family history of psychiatric problems. Following the evaluation at the ED, the patient was admitted to the inpatient psychiatric care unit with suicide precautions. During the initial inpatient evaluation, the patient reported feeling depressed and anxious, as well as having episodic nightmares once every few months due to having witnessed many scenes of death as a firefighter, including a suicide by hanging and violent car wrecks. He reported that, for the previous 3 months, he had been having thoughts about killing himself which was worsened with seeing firearms, razor blades, or pocket knife. He reported that the thoughts of suicide started after witnessing the carnage of a car wreck 3 months before. Earlier in the day, prior to admission, he had held an unloaded gun to his head, which was witnessed by his family and was the ultimate reason the family brought him to the ED. He also reported not being able to relax, feeling on edge, and feeling like he should always be doing something. All potential medical or substance related causes of the patient's psychiatric condition were ruled out. The initial list of differential diagnoses included major depressive disorder (MDD), bipolar disorder, and cyclothymia (due to the reported symptoms of depression and impulsivity). The patient's progress through the initial hospital admission is outlined in Table 2. The patient was ultimately discharged with olanzapine, divalproex extended release, prazosin, clonazepam, and appointments for a therapist and an outpatient psychiatrist.

Two days after being discharged from the inpatient psychiatric care unit, the patient was readmitted with continuing suicidal thoughts which he was unable to control. The patient reported that, during the previous admission, he had told the clinical staff that his symptoms were improving so that could be discharged from the hospital. During the ensuing interview, the patient described in a greater detail the nature of his suicidal thoughts. He reported that he was having sudden onset, intense, recurrent (5-6 times a day), short lasting (a few minutes each), anxiety provoking, unwanted, and distressing thoughts of committing suicide for the past 3 months, since witnessing a car wreck. He reported that he did not believe that his family would be better off without him and that thinking about what his death would do to his wife also caused him significant anxiety and distress. He reported that he rapidly became overwhelmed and panicked with each intense thought of suicide. The patient's social and occupational life was significantly affected due to the obsessive thoughts, becoming more isolated from his family members and skipping work due to fear of triggering suicidal obsessive thoughts. The patient denied any compulsive rituals or repetitive behaviors to reduce the anxiety in relation to obsessive suicidal thoughts. However, he did report trying to block those thoughts by distracting himself, attempting to redirect his thoughts, or seeking reassurance from his family. He expressed a sense of hopelessness and helplessness regarding the intrusive suicidal thoughts. He also reported low self-esteem and negative self-image due to his inability to control those distressing thoughts. He said, as the thoughts continued to return, he did think about acting on those thoughts to make them stop. At the same time, he noted that harming himself was not something that he wanted to do and that he wanted to get help so that he would not actually do so. Once again, all potential medical or substance related causes of the patient's psychiatric condition were ruled out and according to the Diagnostic Statistical Manual of Mental Disorders, 5th ed. (DSM-V), the patient was diagnosed with OCD with obsessive suicidal thoughts without compulsions.

The patient was started on a pharmacotherapy regimen including sertraline 25 mg oral tablet once daily for OCD, PTSD, and MDD, olanzapine 10 mg oral tablet at bedtime for impulse control, and clonazepam 0.5 mg oral tablet three times a day for anxiety. In the ensuing days, the patient was treated with exposure and response prevention (ERP) therapy, an effective cognitive-behavioral therapy for OCD. During the ERP therapy sessions, the patient was instructed to focus on a suicidal thought, report the level of anxiety to the therapist, and avoid calling for an ambulance or calling his wife for reassurance. The patient's anxiety levels were high during the initial sessions of ERP. Motivational interviewing techniques were used to provide encouragement and support in helping the patient continue the therapy. Once the patient's anxiety was decreased and the thought became nondistressing, the patient was encouraged to focus on a different suicidal thought and continue pondering on those thoughts within the ERP therapy regimen, until the thought was no longer distressing. The patient received multiple ERP sessions during the hospitalization, each with different types of suicidal thoughts. Later ERP sessions included cues to more disturbing suicidal thoughts. By the end of the hospitalization, the patient reported a change in his relationship with the obsessive suicidal thoughts and learned that he did not have to engage in suppression or avoidant strategies when those thoughts occurred.

Over the next several days, his sertraline dose was also gradually increased to 150 mg once daily. The patient continued on sertraline, olanzapine, clonazepam, and individual CBT. He reported a decrease in the occurrence of his obsessive suicidal thought, decreased distress when it did appear, and no further need for avoidant behaviors related to the thought. The patient gradually became more verbal, his affect returned to full range, and he actively began participating in group therapy sessions. The clonazepam was tapered off and the patient was discharged on hospital day 8, with sertraline and olanzapine. He was scheduled for follow-up with an individual therapist to help him continue to develop skills to better manage his anxiety and obsessive suicidal thoughts.

## 3. Discussion

The Diagnostics and Statistics Manual of Mental Disorders, 5th ed. (DSM-5), defines suicidal ideation as “thoughts about self-harm, with deliberate consideration or planning of possible techniques of causing one's own death.” Rumination of suicidal ideation in mood disorders is generally mood-congruent and not necessarily experienced as intrusive, distressing, or linked to compulsions [1]. Suicidal obsession on the other hand is characterized by recurrent, persistent, intrusive, and unwanted thoughts about suicide that cause marked anxiety or distress [1, 13, 14]. Factors suggesting OCD related thoughts include ego-dystonicity, absence of past behavior consistent with the thought, presence of avoidance behavior (i.e., avoidance of any tools of suicide), frequent thoughts, high degree of distress, and fair-good insight with strong motivation to seek help [17].

Patients with OCD have been reported to be at a greater risk of suicide than the general population [3–11]. Furthermore, suicidal risk in patients with OCD has been reported to be worsened by comorbid psychiatric conditions or OCD with aggressive or violent obsessions [9–12]. However, it is important to recognize that suicidal thoughts in patients with OCD can be an obsession of self-harm that is distinct from factual suicidal ideations. OCD with ego-dystonic suicidal obsessions have rarely been reported in the literature (see Table 1). Each previously reported case of OCD with suicidal obsessions has been in patients with history of long standing OCD, treatment refractory OCD, or history of suicide attempt (Table 1) [13–15]. The case report by Al-Zaben [13] describes a patient with suicidal obsession in long standing OCD with comorbid depression. A case report by Wetzler et al. [14] described a patient with long standing MDD who developed obsessive thoughts of harming herself following a failed suicide attempt (posttraumatic obsession). In addition, a case report by Watterneck et al. [15] described suicidal obsession in a patient with chronic treatment-resistant OCD with comorbid depression who was successfully treated with methodology described by Foa et al. [18], an evidence-based treatment for OCD. There is also a case report describing ego-dystonic suicidal obsessions occurring as a dose-dependent side-effect of clozapine [16].

The patient described in this case report is unique in that, unlike previously reported cases of OCD with suicidal obsessions, he did not have previous history of any chronic neuropsychiatric diagnoses or any cognitive-behavioral issues. In addition, our patient did not demonstrate any compulsive rituals to reduce his anxiety caused by his obsessive suicidal thoughts. Approximately 25% of OCD patients report distressing obsessions without overt compulsive rituals [19]. The most common comorbid psychiatric diagnoses associated with OCD are depression, anxiety, bipolar affective disorder, and attention deficit hyperactivity disorder [20, 21]. Our patient was assessed for comorbidities and other obsessive-compulsive related disorders (OCRD). He was ultimately diagnosed with new onset symptoms of OCD with suicidal obsessions, with comorbid depression, anxiety, and PTSD, all of which were of new onset. Furthermore, the patient did not have any history of violent acts towards self or others. His exposure to suicide and traumatic death was strictly as a witness while on duty as a first responder. Nevertheless, he developed symptoms of depression and PTSD, as well as obsessive suicidal thoughts.

Although our patient exhibited symptoms consistent with MDD and PTSD, his suicidal thoughts were OCD related, with frequent and intrusive, ego-dystonic, and highly distressing suicidal thoughts that were also associated with avoidance behavior and desperate desire for relief from the illogical, obsessive thoughts. Our patient reported that he actually did not want to harm himself and therefore, the recurrent thoughts of suicide were highly anxiety provoking and distressing. He reported that he did not believe that his family would be better off without him and that thinking about what his death would do to his wife also caused him significant anxiety and distress. He also reported that he rapidly became overwhelmed and panicked with each intense thought of suicide. His suicidal ideations were therefore different in nature than “mood-congruent” suicidal ideations observed in uncontrolled severe MDD patients. In addition, he described that his attempts at suicide (holding an unloaded gun to his head prior to admission and unsuccessfully attempting to hang himself with a towel in the shower while at the hospital) were the result of him being unable to redirect his thoughts away from the obsessive suicidal thoughts, ultimately resulting in him acting out those thoughts. Although his suicidal behavior may stem from poor impulse control, they were not compulsions or behavior stemming from poor insight in believing that his family would be better off without him.

Such an unusual initial presentation of OCD, combined with concurrent symptoms of PTSD and mood disorders (which was the initial chief complaint during the first presentation at the emergency department), posed a significant challenge in the accurate diagnosis of the patient's condition, with potentially catastrophic consequences to the patient's wellbeing. A thorough exploration of the origin of the patient's suicidal thoughts is essential to the diagnosis of OCD with suicidal obsessions.

Evidence-based psychotherapy protocols for separate diagnoses have been utilized successfully as a standard for the treatment of a variety of disorders. To address the challenge that arises with treatment of multiple disorders, a Unified Protocol for Emotional Disorders (UP) was developed, largely due to the growing body of research supporting commonalities in many of these disorders [22]. The CBT techniques used across depression and anxiety converge into the core principle of detecting the unique unwanted thoughts, physical sensations, and emotions, as well as the avoidant strategies and negatively reinforce the failed strategy used by the patient. Ultimately, purposeful exposure to the avoided material becomes a core treatment component for therapy with the anxiety and depressive disorders.

The key issue in our case was determining whether the suicidal thoughts and actions were themselves amenable to exposure and avoidance prevention strategies. The unwanted nature of the thought, the connection of the thought to sympathetic arousal, and the reinforcing compulsive behaviors led to the decision to provide a trial of exposure therapy. The treatment utilized, exposure and response therapy (ERP, a form of CBT), combined with pharmacotherapy with a serotonin reuptake inhibitor (SSRI) plus antipsychotics, as described by Seibell and Hollander [2] for the treatment of OCD, was effective in helping the patient manage the anxiety and distress stemming from his OCD with suicidal obsession.

## 4. Conclusion

In summary, OCD with suicidal obsessions is a rarely reported presentation of OCD. Differentiating between suicidal ideation in the context of psychiatric illnesses and suicidal obsessions in OCD is critical to ensuring accurate diagnosis of the patient's condition, as well as timely provision of most appropriate multidisciplinary treatment. As described in this case report, new onset OCD with suicide obsession should not be neglected in the differential in those with newly developing symptoms of OCD and other comorbid psychiatric conditions, especially in those with history of exposure to scenes of suicide and death in personal or professional setting. Patients presenting with suicidal ideation and diagnosed with OCD must undergo a meticulous risk assessment to differentiate suicidal obsession from suicidal ideations, especially when there are other comorbid psychiatric conditions. Furthermore, it is important to assess suicidal ideations in OCD patients with obsessive thoughts of violence, as they are at a higher risk of self-harm [12]. The ability to accurately separate the obsession is critical to provide effective treatment. Therefore, a thorough exploration of the origin of the patient's suicidal thoughts is essential to the accurate diagnosis and timely provision of optimal multidisciplinary treatment of OCD with suicidal obsessions. In the case of our patient, ERP combined with sertraline and olanzapine, which matches the recommended standard of care for OCD with CBT, SSRI, and antipsychotic [2, 23, 24], was effective in the management of his symptoms of OCD with suicidal obsession.

## Figures and Tables

**Table 1:** Previously published case reports on OCD with suicidal obsession.

Report	Age	Sex	History	Comments
Al-Zaben [13]	54	Female	Long history of MDD, long history of OCD	OCD symptoms were improved with paroxetine and cognitive restructuring.

Wetzler et al. [14]	29	Female	Long history of MDD, suicide attempt with posttraumatic obsession	Depression was improved with ECT and citalopram. OCD improved with gradual exposure therapy.

Wetterneck et al. [15]	40s	Male	MDD, chronic treatment resistant OCD	Improved after treatment with Foa et al.'s protocol on exposure and ritual/response prevention (ERP), an evidence-based treatment for OCD.

Aukst-Margetić et al. [16]	54	Male	Bipolar disorder	Developed ego-dystonic suicidal obsession with clozapine in a dose dependent manner. Improved with switch to a different atypical antipsychotic.

**Table 2:** Progression of events during the first and second admissions.

Hospital day	Key events
*First hospitalization*	
Day 1	(i) Initial differential diagnoses based on the initial interview and psychological testing (a) Major depressive disorder (b) Bipolar disorder (c) Cyclothymic disorder (mood fluctuation with impulsivity). (ii) Started citalopram 10 mg PO once daily for depression. (iii) Started gabapentin 100 mg PO three times daily for anxiety.

Day 2	(i) Patient reports improvement in his depression and anxiety. (ii) Patient unsuccessfully seeks to be discharged.

Day 3	(i) Patient reports having nightmares about him hanging himself with an extension cord in the backyard of his home. (ii) Patient reports having intrusive, recurrent, distressing thoughts about suicide. (iii) Gabapentin 300 mg PO three times a day replaced with carbamazepine 200 mg PO twice a day for impulsivity.

Day 4	(i) Patient upset at his family for not consenting to his discharge. (ii) Patient reports that while in his room to “cool off” having another sudden intrusive thought of hanging himself and unsuccessfully attempts to hang himself in the shower. (iii) Patient treated for a scalp laceration resulting from the unsuccessful suicide attempt but was found to be not otherwise physically harmed.

Day 5	(i) Patient reevaluated following the suicide attempt to clarify the diagnosis. (ii) Patient reports having prominent symptoms reflective of PTSD for 2 months (a) Recurrent flashbacks of multiple traumatic scenes witnessed while working as a firefighter (b) Patient reports having recurrent images of others hanging (c) Patient reports feeling like he is on edge, feeling physically ill, and having avoided work as a firefighter to prevent having recurrent images of death. (iii) Started prazosin 1 mg PO at bedtime for PTSD. (iv) Bipolar disorder and cyclothymic disorder ruled out after continuing assessment. (v) Discontinued carbamazepine 200 mg PO twice a day.

Day 6	(i) Patient reports continuing recurrent suicidal thoughts which causes him significant distress. (ii) Patient attempts to clear his head of those intrusive thoughts by repeatedly punching his own head. (iii) Patient further screened for other possible diagnoses including self-injurious behavior and obsessive suicidal thoughts. (iv) Starting a trial of divalproex sodium 500 mg extended release PO twice a day and olanzapine 10 mg oral tablet at bedtime for impulsive thoughts.

Day 7	(i) Patient remains worried about intrusive suicidal thoughts and expresses hopelessness with regard to keeping his intrusive suicidal thoughts under control. (ii) Discontinued citalopram 10 mg oral tablet once daily due to mood lability and persisting suicidal ideations.

Day 8	(i) Patient reports decreased frequency of intrusive suicidal thoughts. (ii) Started clonazepam 0.5 mg oral tablet three times a day for anxiety.

Days 9–12	(i) Patient reports improvement with intrusive suicidal thoughts and denies suicidal intent. (ii) Patient discharged with (a) olanzapine 10 mg PO once daily, (b) divalproex sodium 500 mg extended release PO twice a day, (c) prazosin 1 mg PO at bedtime for PTSD, (d) clonazepam 0.5 mg PO three times a day, (e) new appointment for therapy, (f) follow-up appointment for psychiatry.

*Second hospitalization*	
Day 1	(i) Readmitted within 48 hours after discharge with recurrent intrusive suicidal thoughts. (ii) EEG r/o dysrhythmias and epileptiform activity.

Day 2	(i) The patient describes suicidal thoughts as unwanted and intrusive and happening “about 5-6 times a day.” These obsessive suicidal thoughts started suddenly three months before, after he witnessed a scene of a car wreck. They are short in duration “not very long, a few minutes” but are “very strong” and disturbing to him. He reports becoming overwhelmed and panicked. He tries to block them “by thinking of anything else…trying to distract myself.” That only works for a while and then the pattern repeats. He said that as they continue to return, he gets more upset and begins to think about “hanging myself” to make them go away. (ii) The patient denies any previous suicidal thoughts even though he had been feeling depressed, prior to seeing the carnage of the car wreck. (iii) The patient denies thinking that his family would be better off without him. He reports that he has not given any possessions away; he has not written a suicide note; he has not done anything else to prepare for death (will, insurance, property, or practicing). (iv) The patient notes that he does not want to kill himself and one reason is the effect on his wife and family adding, “That's why I let them know I have these thoughts.” He noted, “I can control it sometimes but they keep coming back.” (v) He reports he is hopeful about the future, “I hope I can get better…isn't there a medication that can help?” (vi) The patient is stable with depression. He reports extreme anxiety due to obsessive suicidal thoughts. He denies any psychotic symptoms and also denies any homicidal ideations. (vii) The patient is diagnosed with OCD based on DSM-5 criteria. (viii) The patient agreed to start exposure and response prevention (ERP) therapy. (ix) Medications (a) Stopped divalproex sodium ER (b) Sertraline 50 mg PO once daily (c) Clonazepam 0.5 mg PO twice daily (d) Olanzapine to 5 mg PO twice daily

Day 3	(i) The patient participates in additional ERP therapy. (ii) The patient states, “I am hopeful.” (iii) The patient verbalizes improvement in obsessive suicidal thoughts with decreased anxiety and distress stemming from suicidal thoughts. (iv) Medications (a) Increased sertraline to 100 mg PO once daily (b) Clonazepam 0.5 mg PO twice daily (c) Olanzapine to 5 mg PO twice daily

Day 4	(i) The patient participates in more prolonged exposure therapy. (ii) The patient reports overall improvement in his mood and less anxiety about his obsessive suicidal thoughts. He rates his anxiety at 4 out of 10, with 10 being the maximum. (iii) Medications (a) Increased sertraline to 150 mg PO once daily (b) Clonazepam 0.5 mg PO twice daily (c) Olanzapine to 5 mg PO twice daily

Day 5–8	(i) The patient reports being able to manage his obsessive suicidal thoughts and rates his anxiety regarding suicidal thoughts at 0 out of 10. (ii) He then reports no suicidal thoughts three days in a row. He continuously participates in therapy. (iii) Family members notice improvement with patient's anxiety and depression. The patient is discharged home with psychiatry and psychology follow-up as out-patient. (iv) Discharge medications (a) Sertraline 150 mg PO once daily (b) Olanzapine to 5 mg PO twice daily (c) Clonazepam tapered off
